# How Vision Loss Affects Different Psychological Outcomes Among Older Adults?

**DOI:** 10.1093/geroni/igaf122.307

**Published:** 2025-12-31

**Authors:** Ya-Han Chang, Silvia Sörensen

**Affiliations:** University of Rochester, Rochester, New York, United States; University of Rochester Warner School of Education and Human Development, Rochester, New York, United States

## Abstract

**Background:**

Loneliness (Alma et al., 2011; Mick et al., 2018), anxiety and depression (Evans et al., 2007) are more prevalent among visually impaired (VI) than sighted older adults. Individuals’ emotional response to vision loss (ERVL) may be important to determining these outcomes.

**Methods:**

We used baseline data from adults with macular degeneration (N = 210, Mean Age = 80.1) participating in a depression prevention intervention (Sörensen et al., 2015). ERVL was assessed using 12 items of NEI-VFQ items (RAND, 1996) focused on emotions and perceptions of living with VI (α=.904). We used MANCOVA to test the effect of ERVL on loneliness, depression, anxiety, worries about future care, and perceived stress.

**Results:**

The multivariate effect of ERVL on the combined psychological outcomes was significant (Wilks’ Lambda = .249, p < .01, η² = .242). ERVL was significantly associated with loneliness (p<.05, η² = .265), depression (p<.05, η² = .290), and perceived stress level (p<.001, η² = .335). ERVL was not associated with anxiety (p>.05, η² = .224 ) and worries about future care (p>.05, η² = .257).

**Conclusion:**

How individuals perceive and respond to challenging situations can affect psychological outcomes, supporting the idea that stress appraisal is an important aspect of coping with vision loss.

